# Antibacterial, Resistance Modulation, Anti-Biofilm Formation, and Efflux Pump Inhibition Properties of *Loeseneriella africana* (Willd.) N. Halle (Celastraceae) Stem Extract and Its Constituents

**DOI:** 10.3390/microorganisms12010007

**Published:** 2023-12-19

**Authors:** Daniel Anokwah, Evelyn Asante-Kwatia, Jonathan Asante, Daniel Obeng-Mensah, Cynthia Amaning Danquah, Isaac Kingsley Amponsah, Elvis Ofori Ameyaw, Robert Peter Biney, Ernest Obese, Lukas Oberer, Daniel Gyamfi Amoako, Akebe Luther King Abia, Abraham Yeboah Mensah

**Affiliations:** 1 School of Pharmacy and Pharmaceutical Sciences, College of Health and Allied Sciences, University of Cape Coast, PMB, Cape Coast, Ghana; jonathan.asante@ucc.edu.gh (J.A.); daniel.obeng-mensah@ucc.edu.gh (D.O.-M.); eameyaw@ucc.edu.gh (E.O.A.); robert.biney@ucc.edu.gh (R.P.B.); ernest.obese@ucc.edu.gh (E.O.); 2 Department of Pharmacognosy, Faculty of Pharmacy and Pharmaceutical Sciences, Kwame Nkrumah University of Science and Technology, PMB, Kumasi, Ghana; eamireku@knust.edu.gh (E.A.-K.); akila.amponsah@gmail.com (I.K.A.); aymensah@yahoo.com (A.Y.M.); 3 Department of Pharmacology, Faculty of Pharmacy and Pharmaceutical Sciences, Kwame Nkrumah University of Science and Technology, PMB, Kumasi, Ghana; cadanq@yahoo.com; 4 Novartis Institutes for BioMedical Research, CH-4056 Basel, Switzerland; lukas.oberer@bluewin.ch; 5 Antimicrobial Research Unit, College of Health Sciences, University of KwaZulu-Natal, Durban 4001, South Africa; amoakodg@gmail.com (D.G.A.); lutherkinga@yahoo.fr (A.L.K.A.); 6 Environmental Research Foundation, Westville 3630, South Africa

**Keywords:** *Loeseneriella africana*, phytochemical screening, friedelane-1,3-dione, β-sitosterol, chromatography

## Abstract

This study investigated the antibacterial, resistance modulation, biofilm inhibition, and efflux pump inhibition potentials of *Loeseneriella africana* stem extract and its constituents. The antimicrobial activity was investigated by the high-throughput spot culture growth inhibition (HT-SPOTi) and broth microdilution assays. The resistance modulation activity was investigated using the anti-biofilm formation and efflux pump inhibition assays. Purification of the extract was carried out by chromatographic methods, and the isolated compounds were characterized based on nuclear magnetic resonance, Fourier transform infrared and mass spectrometry spectral data and comparison with published literature. The whole extract, methanol, ethyl acetate, and pet-ether fractions of *L. africana* all showed antibacterial activity against the test bacteria with MICs ranging from 62.5 to 500.0 µg/mL The whole extract demonstrated resistance modulation effect through strong biofilm inhibition and efflux pump inhibition activities against *S. aureus* ATCC 25923, *E. coli* ATCC 25922 and *P. aeruginosa* ATCC 27853. Chromatographic fractionation of the ethyl acetate fraction resulted in the isolation of a triterpenoid (4S,4αS,6αR,6βS,8αS,12αS,12βR,14αS,14βR)-4,4α,6β,8α,11,11,12β,14α-Octamethyloctadecahydropicene-1,3(2H,4H)-dione) and a phytosterol (β-sitosterol). These compounds showed antibacterial activity against susceptible bacteria at a MIC range of 31–125 µg/mL and potentiated the antibacterial activity of amoxicillin (at ¼ MIC of compounds) against *E. coli* and *P. aeruginosa* with modulation factors of 32 and 10, respectively. These compounds also demonstrated good anti-biofilm formation effect at a concentration range of 3–100 µg/mL, and bacterial efflux pump inhibition activity at ½ MIC and ¼ MIC against *E. coli* and *P. aeruginosa*. *Loeseneriella africana* stem bark extracts and constituents elicit considerable antibacterial, resistance modulation, and biofilm and efflux pump inhibition activities. The results justify the indigenous uses of *L. africana* for managing microbial infections.

## 1. Introduction

The evolution of multidrug-resistant (MDR) bacteria remains a global health menace due to the consequent increased morbidity and mortality [1]. Globally, the treatment of infectious diseases with the currently available antibiotics is becoming increasingly difficult due to the development of multidrug resistance mechanisms by bacteria [2]. Unfortunately, this problem is exacerbated by the rapidly depleting pipeline of new antibiotics, necessitating an urgent search for new antimicrobial agents [3].

Bacterial biofilm formation and the overexpression of efflux pumps have been shown as major resistance mechanisms [4,5]. In comparison to their planktonic counterparts, bacteria in biofilms are about 1000 times more resistant to antibiotics. The biofilm matrix decreases permeability and allows type IV secretion systems (complex transmembrane secretion proteins) of bacteria to mediate horizontal gene transfer, which facilitates antibiotic resistance among bacteria through adaptation to environmental changes [5,6]. When formed, the biofilm matrix facilitates the action of antibiotic-modifying enzymes, and the expression of multidrug efflux pumps in the bacterial cells [6,7,8]. Overexpression of efflux proteins by bacteria allows them to extrude several antibiotics to their exterior, preventing antibiotics from reaching their therapeutic concentration, thereby rendering them ineffective [5,6]. Therefore, developing medicines that prevent biofilm formation and combine chemical efflux pump inhibitors with existing antibiotics is a potential strategy for fighting antimicrobial resistance.

Medicinal plants have been demonstrated to be rich sources of secondary metabolites with potential uses as therapeutic agents. These secondary metabolites possess diverse chemical structures and mechanisms of action which may be valuable in developing new treatment against resistant infections [9,10]. Furthermore, medicinal plants are widely used in many communities due to their availability and favourable safety profile [11].

As part of a continuing effort to explore tropical medicinal plants as sources of antimicrobial agents, the antibacterial activity, anti-biofilm, efflux pump inhibitory and resistance modulation potentials of the crude extract, fractions and some constituents of the stem of *Loeseneriella africana* were investigated in this study. *Loeseneriella africana* (Willd.) N. Halle is among 16 known species of the genus *Loeseneriella* of the family *Celestraceae*. *Loeseneriella africana* is a tough, flexible and durable liane or scandent shrub with widespread distribution in West Africa, South Africa, Sri Lanka, India, Laos, and Myanmar [12,13].

*Loeseneriella africana* is used in African folklore medicine to treat inflammatory and infectious diseases. In Ghana, the stem decoction is used for treating malaria, wounds, oedema, menstrual pains, and in combination with other plants for infectious diseases and hypertension [14]. Previous reports show that *Loeseneriella africana* and related species have antidiabetic and hypolipidaemic, analgesic, anti-inflammatory, antipyretic, antioxidant, antidiarrheal and antinuclear activities [13,14]. Previous phytochemical profiling of the stem and leaves of *L. africana* has used HPLC-MS detected flavonoids including rutin, p-coumaric acid, isoquercitrin, quercetin, quercitrin, ferulic acids and gentisic as bioactive molecules, which could be responsible for the anti-inflammatory and antioxidant potentials of the plant [13]. However, little is known about its antimicrobial properties, prompting this study. Therefore, the objective of the study is to determine the antibacterial, resistance modulation, biofilm inhibition, and efflux pump inhibition potential of *L. africana* stem and its constituents as possible alternatives in mitigating microbial resistance through direct antimicrobial activity or resistance modulation activity.

## 2. Materials and Methods

### 2.1. Drugs and Chemicals

Solvents were supplied by BDH Laboratory Supplies, London, UK. Silica gel 70–230 mesh and aluminum sheet TLC silica gel 60 F_254_ were obtained from Merck KGaA (Darmstadt, Germany), and amoxicillin was purchased from Phyto-Riker, Accra, Ghana.

### 2.2. Bacterial Strains and Inoculum Standardization

Clinical bacterial strains (*Streptococcus pyogenes*, *Vibrio cholerae*, *Salmonella typhi*, *Klebsiella pneumoniae*) were obtained from the pharmaceutical microbiology laboratory, Kwame Nkrumah University of Science and Technology (KNUST). Also, American type culture collection (ATCC) bacteria strains (*Enterococcus faecalis* ATCC 29212, *Escherichia coli* ATCC 25922, *Staphylococcus aureus* ATCC 25923, *Proteus mirabilis* ATCC 12453 and *Pseudomonas aeruginosa* ATCC 27853) were supplied by the Department of Pharmacology, KNUST, Kumasi, Ghana.

Overnight broth cultures of test organisms were used to create a standardized bacteria culture. The organisms were standardized through serial dilution in sterile normal saline (0.9 percent NaCl, *w*/*v*) to achieve about 1.5 × 10^8^ CFU/mL (equivalent to 0.5 McFarland standard).

### 2.3. General Experimental Procedures

Column chromatography (CC) was carried out using silica gel 60 (70−230 mesh). Thin layer chromatography (TLC) was conducted using pre-coated silica gel 60 plates (0.25 mm). Separated compounds were visualized by spraying with vanillin-sulphuric acid spray reagent followed by heating. Mass spectrometry on isolated compounds was carried out with high-resolution electrospray ionization mass spectrometry (HR-ESI-MS). The instrument used was Bruker MaXis 288882.20181 (LC-QTOF) supplied by the Bruker Corporation, Billerica, MA, USA. Nuclear magnetic resonance (NMR) data were recorded on a Bruker 500 MHz Advance III HD NMR spectrometer (Bruker Corporation, Billerica, MA, USA), operating at 500 MHz (^1^H) and 125 MHz (^13^C) with deuterated methanol (CD_3_OD) as a solvent. The chemical shifts were expressed as parts per million (ppm) with tetramethylsilane (TMS) as the internal standard and the coupling constants (*J*) were measured in hertz (Hz). Functional groups in the isolated compounds were identified using the Fourier transform-infrared spectrometry (FT-IR) performed on a Perkin Elmer device (UATR Spectrum Two Series) to obtain infrared (IR) spectra over a 4000–350 cm^−1^ wave number range.

### 2.4. Plant Material Collection

The stem of *L. africana* was harvested from the wild at Kwahu Asakraka village in the eastern region of Ghana (06°37.356′ N/000°41.396′ W) in November 2017. The plant material was authenticated by Mr. Clifford Asare of the Department of Herbal Medicine, KNUST, Kumasi, Ghana, and a voucher specimen (KNUST/HM/2017/SB017) was reserved in the department’s herbarium.

### 2.5. Preliminary Phytochemical Screening

Secondary metabolites such as tannins, flavonoids, triterpenoids, and glycosides were detected in the *L. africana* stem powder using simple qualitative phytochemical screening methods [15].

### 2.6. Preparation of Extracts and Fractions

After washing under running water, the stem was cut into smaller sizes suitable for milling, and air-dried for 10 days. The dried plant material was powdered with a hammer mill into coarse powder of about 3 kg and was extracted with methanol for 6 h using a Soxhlet extractor. The mother liquor was concentrated on a rotavapor under reduced pressure and further dried at 65 °C overnight to obtain a brown solid extract (6.4% yield) referred to as LA or “the whole extract” in this report. About 120 g of LA was adsorbed onto silica gel (70–230 mesh size) successively partitioned with petroleum ether (pet-ether), ethyl acetate (EtOAc) and methanol (MeOH) to afford pet-ether (LAPE, 7.3 g), EtOAc (LAEt, 27.9 g) and MeOH (LAM, 82.6 g) fractions. A desiccator was used to store the extract and fractions until required for use.

### 2.7. Isolation and Characterization of Phytoconstituents

**Figure 1:**
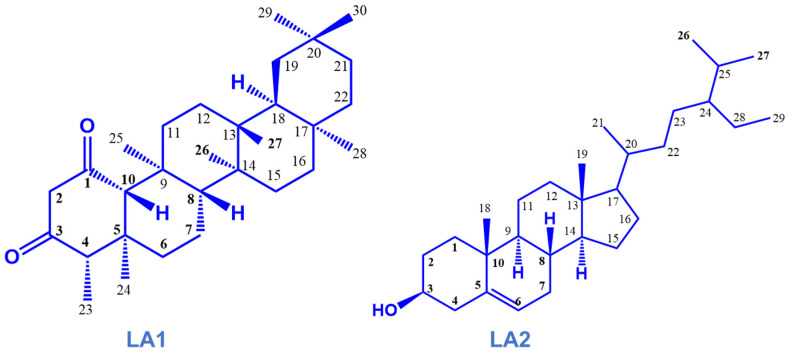
Isolated compounds from the stem bark of *Loesenoriella africana*.

About 25 g of the ethyl acetate fraction (LAEt) was mounted and purified by column chromatography (CC) [16]. Ethyl acetate was used to reconstitute the extract, adsorbed onto silica gel 60, and dry-packed onto the stationary phase (silica gel, 70−230 mesh). The solvents (pet-ether, EtOAc and MeOH) were used as the mobile phase by gradient elution and the eluates were monitored using TLC. Two compounds were obtained and characterized by comparing their ^1^H, ^13^C NMR and mass spectral data with published data (Figure 1). Details of the isolation and characterization are presented in Figure S1 (Supplementary Materials).

### 2.8. Antimicrobial Testing

#### 2.8.1. Evaluation of Antibacterial Activity of Crude Extracts and Fractions

The crude MeOH extract and major fractions were tested for antibacterial activity using the high-throughput spot culture growth inhibition assay (HT-SPOTi) as previously described [17]. Briefly, a twofold serial dilution of a stock solution of the extracts, prepared in dimethyl sulphoxide (2% DMSO), was carried out in a PCR half-skirted plate to give a concentration range of 0.49–500.00 µg/mL. Then, 2 µL of each dilution was allotted into corresponding wells of a 96-well plate. After that, 200 µL of molten agar was added to wells containing the test samples and swirled to mix thoroughly. After setting, the wells were spotted with 2 µL of standardized suspension of the test microorganisms and allowed to stand for 20 min to enable diffusion of the test sample into the agar. Wells containing only media and bacteria were used as a negative control and wells containing media, bacteria and amoxicillin were used as a positive control. The plates were sealed and incubated at 37 °C for 24 h. Visual examination for comparison with the control wells was used to determine the presence or absence of growth.

#### 2.8.2. Determination of the Antibacterial Activity and Antibiotic Modulation Effect of Isolated Compounds

The isolated compounds were investigated for their antibacterial activity by the broth microdilution method against selected Gram-negative (*P. aeruginosa* ATCC 27853 and *E. coli* ATCC 25922) and Gram-positive (*S. aureus* ATCC 25923 and *E. faecalis* ATCC 29212) bacteria according to the World Health Organization (WHO) priority [18]. Briefly, respective wells of a 96-well microplate were filled with 100 µL of nutrient broth (NB) and corresponding volumes of compounds reconstituted in DMSO (2%) to obtain a concentration range between 7.8 and 500.0 µg/mL. Then, 20 µL of test organisms (approximately 10^8^ CFU/mL) were inoculated and the plates incubated at 37 °C for 24 h. The presence or absence of growth was determined using the visual colour change of 3-(4,5-dimethylthiazol-2-yl)-2,5-diphenyl-2H-tetrazolium bromide (MTT) (0.125% *w*/*v* thiazoyl blue tetrazolium bromide) within 30 min of addition to the wells.

For the resistance modulating test, the MIC of the standard drug (amoxicillin) was determined in the presence of 1/4 MICs of the phytoconstituents against *E. coli* and *P. aeruginosa*. The resistance modulation activity of the phytoconstituent on the MIC of the standard drug was determined by the modulation factor (MF). The estimation was done using the formula: MF = MIC (antibiotic)/MIC (antibiotic + modulator) [19].

#### 2.8.3. Biofilm Inhibition Assay

The whole extract (LA) and the two compounds were screened for their effect on the biofilm formation by *E. coli* ATCC 25922, *P. aeruginosa* ATCC 27853 and *S. aureus* ATCC 25923 using the crystal violet retention method as previously described [16,20]. Briefly, 180 µL of microorganisms freshly cultured in tryptone soy broth (TSB) was pipetted into corresponding wells of a flat-bottom 96-well polystyrene microtiter plate. Concurrently, 20 µL of test extract (15–500 µg/mL) or compounds (3.1–100.0 µg/mL) prepared in TSB was added and the plates incubated at 37 °C for 24 h (subsequently referred to as the test). Wells containing only media and bacteria were used as control wells for the assay. The experiment was conducted in quadruplicate. After 24 h of incubation, the supernatant was aspirated, and the planktonic cells were washed off with phosphate buffer saline (PBS, pH 7.2). The adherent biofilms were fixed by drying the plates in the incubator at 50 °C for 30 min. The biofilms were then stained with 200 μL of 0.1% *w*/*v* crystal violet_(aq)_ (CV) dye for 10 min at 26 °C. Excess dye was aspirated and the wells were gently washed three times with sterile water for injection. Finally, the stain bound to the biofilms in each well was solubilized with 200 µL of 95% ethanol and allowed to stand for 10 min. The differential staining absorbance was measured at 600 nm using a microtiter plate reader (Biotek Synergy H1 Hybrid Multi-Mode Reader: 271230 supplied by Agilent Technologies, Santa Clara, CA, USA). The mean absorbance of the samples was determined, and the percentage inhibition of biofilm was calculated as: Percentage biofilm inhibition (%) = [(control OD_600_ − Test OD_600_)/control OD_600_] × 100.

#### 2.8.4. Efflux Pump Inhibition Assay

The extract (LA) and compounds were investigated for their effect on the efflux pump activity of bacterial cells according to previously published protocols [19,21]. The organisms (*E. coli* ATCC 25922, *P. aeruginosa* ATCC 27853 and *S. aureus* ATCC 25923) were grown to the mid-log phase in the nutrient broth (OD_600_ adjusted to 0.4) and collected by centrifugation (3000 rpm for 15 min at 4 °C). The supernatant was discarded and the cells were re-suspended twice in sterile PBS at pH 7.4 and diluted to a final OD_600_ of 0.5.

Aliquots of the bacterial cell suspension in PBS were added to filter sterilized glucose (mixed to a final concentration of 0.4% *v*/*v*) and pipetted into corresponding wells of a 96-well microtiter plate. The plates were vortexed at room temperature to distribute the cells uniformly. Ethidium bromide (EtBr) was added to a final concentration of 0.5 mg/L and different volumes of the extract/compound (1/2 or 1/4 MIC) were added to corresponding wells and the fluorescence was measured over 60 min at 3 min intervals using excitation and emission wavelengths of 530 nm and 585 nm, respectively. Chlorpromazine and verapamil, known efflux pump inhibitors (EPI), were included as comparative probes and a drug-free culture as the negative control. The ability of the test substance to enhance the accumulation of EtBr (a substrate for efflux pumps) is considered as an efflux pump inhibition effect [18].

## 3. Results

### 3.1. Preliminary Phytochemical Investigation of the Stem of L. africana

Preliminary phytochemical analyses of the dried powdered *L. africana* stem confirmed the existence of many secondary metabolite groups, including triterpenoids and phytosterols (Table 1).

### 3.2. Isolated Compounds from the Stem of L. africana

Chromatographic fractionation and purification of the bioactive EtOAc fraction led to the isolation of two compounds based on their ^1^H and ^13^C NMR, MS and FTIR data. The white amorphous crystals (compound **LA1**) were identified to be friedelane-1,3-dione triterpenoid, characterized as 4S,4αS,6αR,6βS,8αS,12αS,12βR,14αS,14βR-4,4α,6β,8α,11,11,12β,14α Octamethyloctadecahydropicene-1,3(2H,4H)-dione. The white amorphous powder (compound **LA2**) was identified as a phytosterol, characterized as β -sitosterol (Figure 1). The ^1^H NMR (600 MHz in CDCl_3_) and ^13^C (125 MHz in CDCl_3_) NMR data are shown in Table 2. Further spectral data and some physicochemical constants of the compounds are available in the Supplementary Materials, Figures S2–S4.

### 3.3. Antibacterial Activity of L. africana Stem Extract, Major Fractions and Isolated Compounds

In the HT-SPOTi assay, the methanol-chloroform (4:1) whole extract, petroleum ether, ethyl acetate, and methanol fractions inhibited growth of Gram-positive and Gram-negative bacteria to varying degrees. The MIC for susceptible bacteria ranged between 125 and 500 µg/mL depending on the bacteria and solvent extract (Table 3). The MICs of the extracted compounds from *L. africana* stem are shown in Table 4.

### 3.4. Antibiotic Modulation Effect of Isolated Compounds from L. africana Stem

The isolated compounds were tested against *E. coli* and *P. aeruginosa* for an antibacterial resistance modulatory effect with amoxicillin at sub-inhibitory concentrations (1/4 MIC). In the broth dilution assay (Table 5), the MIC values of amoxicillin against *E. coli* (20 µg/mL) and *P. aeruginosa* (>320 µg/mL) were higher than the clinical breakpoint (8 µg/mL), indicating possible resistance.

### 3.5. Biofilm Inhibitory Effect of L. africana Stem Extract and Isolated Compounds

**Figure 2:**
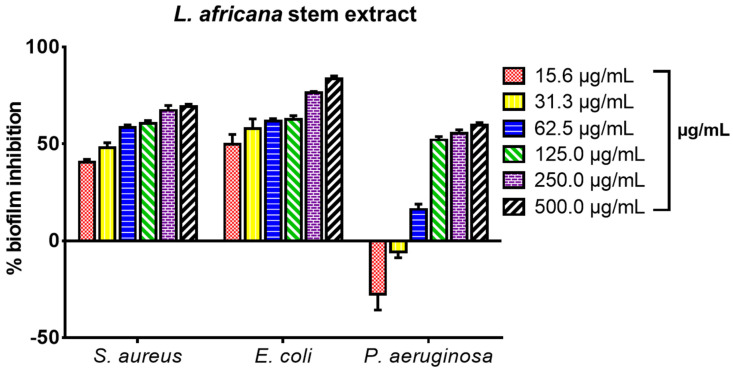
Biofilm formation inhibitory effect of the stem extract of *L. africana* against *S. aureus* ATCC 25923, *E. coli* ATCC 29212, and *P. aeruginosa* ATCC 27853 expressed as percentage biofilm inhibition; values recorded as mean ± SEM (*n* = 3); *p* < 0.05.

The extract inhibited biofilm formation in a concentration-dependent manner between 15.6 and 500.0 µg/mL. The strongest biofilm inhibition effect was observed against *E. coli*, followed by *S. aureus* and *P. aeruginosa* (Figure 2). The percentage biofilm inhibition ranged between 40 and 59% for *S. aureus* at 15.0–62.5 µg/mL, and 49–77% for *E. coli* at 15–250 µg/mL. For *P. aeruginosa*, the percentage biofilm inhibition ranged between 15 and 56% at a sub-inhibitory concentration of 62.5–250 µg/mL whereas at concentrations between 15 and 31 µg/mL, LA enhanced the biofilm-forming capacity of *P. aeruginosa.*

**Figure 3:**
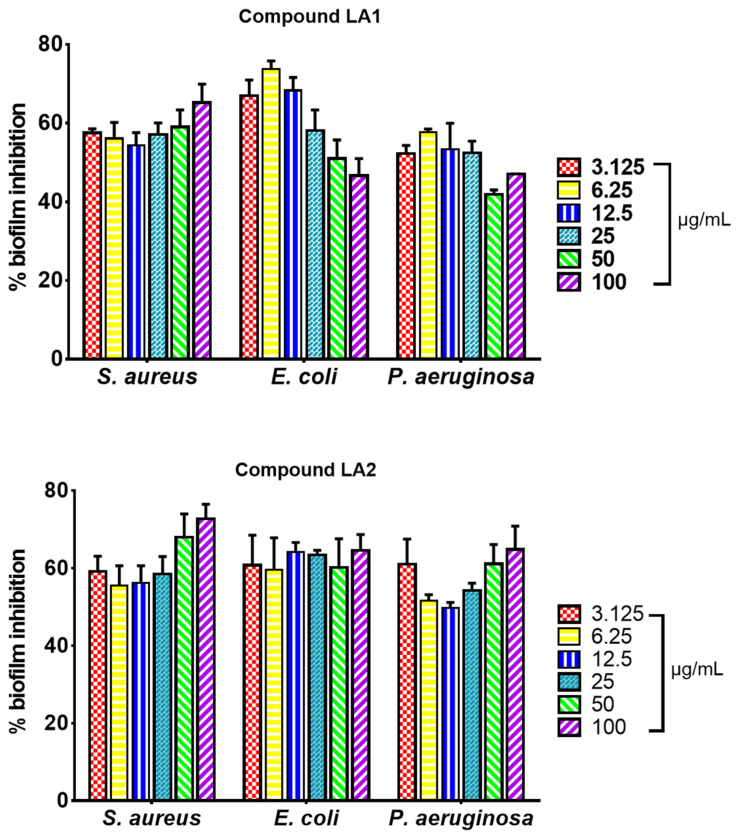
Biofilm formation inhibitory effect of isolated compounds **LA1** and **LA2** on bacteria biofilm formation in *S. aureus* ATCC 25923, *E. coli* ATCC 29212, and *P. aeruginosa* ATCC 27853 expressed as percentage biofilm inhibition; values recorded as mean ± SEM (*n* = 3); *p* < 0.05.

The effect of triterpenoid compounds **LA1** and **LA2** (3.1–100.0 µg/mL) on biofilm formation in *S. aureus*, *E. coli* and *P. aeruginosa* is presented on Figure 3. **LA1** showed good antibiofilm activity against *S. aureus* (54–65%) and *E. coli* (47–74%) whereas it showed low to good activity against *P. aeruginosa* (42–57%). **LA2** demonstrated good antibiofilm activity against *S. aureus* (56–72%), *E. coli* (60–65%) and *P. aeruginosa* (50–65%).

### 3.6. Effect of L. africana Stem Extract and Isolated Compounds on Ethidium Bacterial Efflux Pump

**Figure 4:**
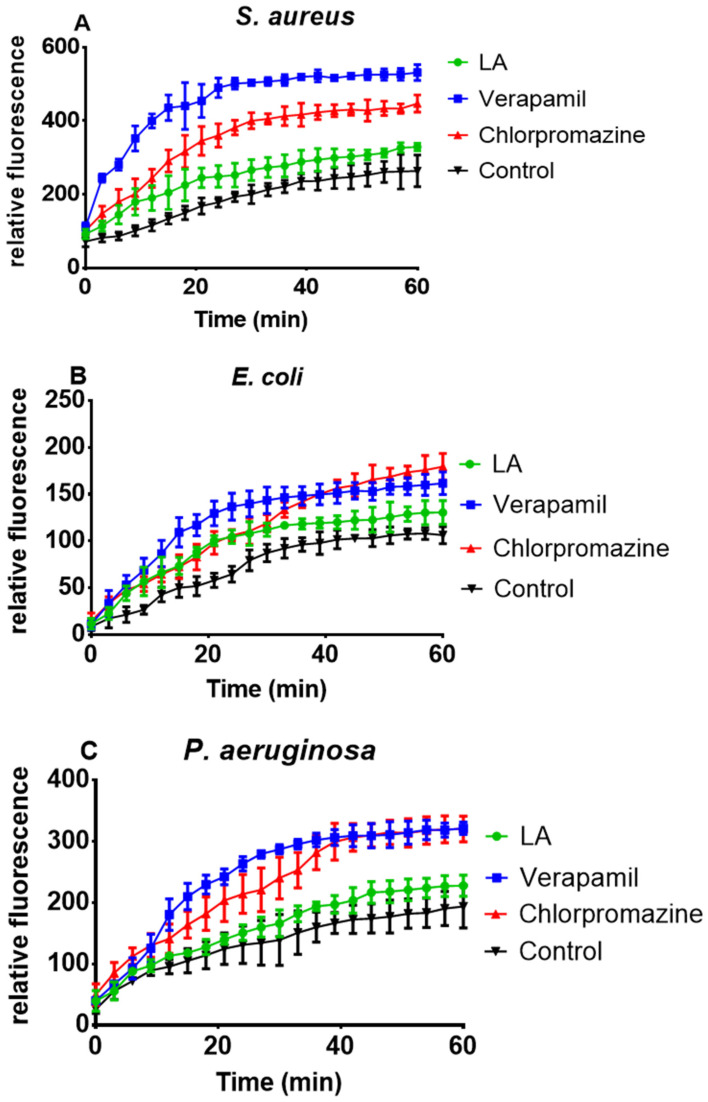
Effect of *L. africana* whole extract (LA) at ½ MIC concentration on EtBr accumulation in *S. aureus* (**A**), *E. coli* (**B**) and *P. aeruginosa* (**C**).

Figure 4 depicts the accumulation behavior of the extract (LA) in *S. aureus*, *E. coli*, and *P. aeruginosa* over 60 min compared to the two standard EPIs, verapamil (VP) and chlorpromazine (CP). According to the results, the crude extract (LA) inhibited efflux pumps, resulting in EtBr fluorescence, whereas both standard EPIs were more effective in causing higher EtBr accumulation in the bacterial cells (*S. aureus*, *E. coli*, and *P. aeruginosa*) than the test extract measured over 60 min (Figure 4A–C).

**Figure 5:**
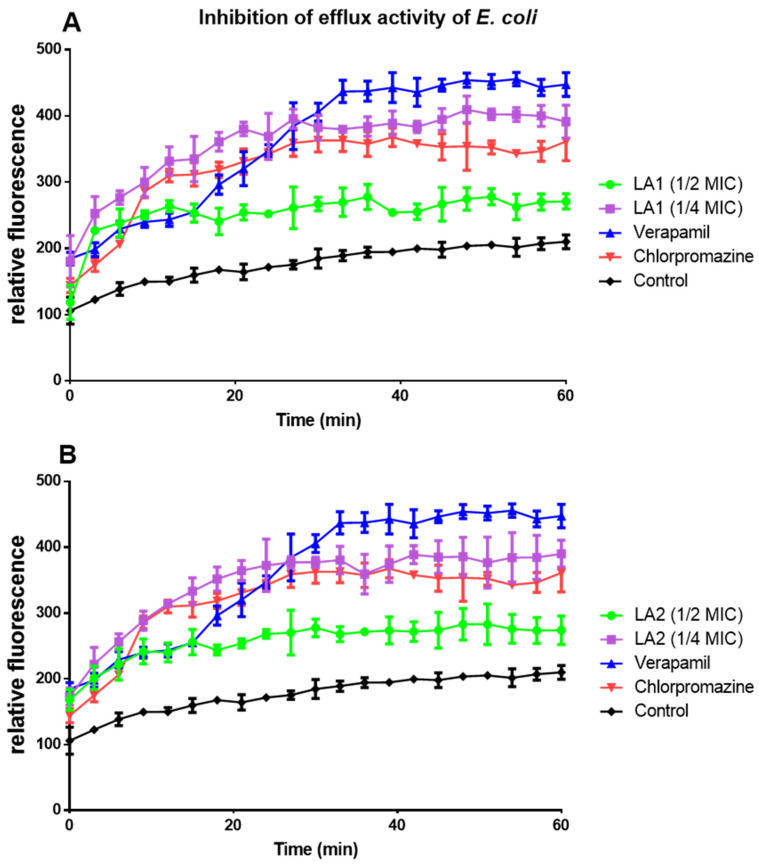
The effect of compounds **LA1** (**A**) and **LA2** (**B**) at ½ and ¼ MICs on the intracellular accumulation of EtBr in *E. coli*.

**Figure 6:**
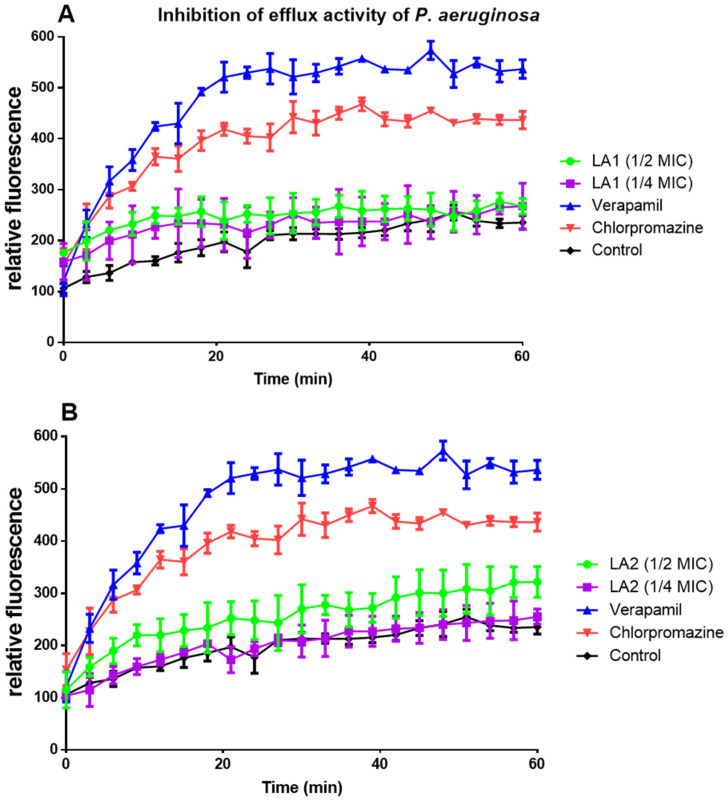
The effect of compounds **LA1** (**A**) and **LA2** (**B**) at ½ and 1/4 MICs on the intracellular accumulation of EtBr in *P. aeruginosa*.

**LA1** and **LA2** acted on efflux pumps by increasing ethidium bromide accumulation in *E. coli* (Figure 5A,B) and *P. aeruginosa* (Figure 6A,B).

The efflux pump inhibition activity of the compounds against *E. coli* (Table 6) and *P. aeruginosa* (Table 7) was calculated every 15 min as a percentage inhibition over the negative control and compared to the standard drugs (verapamil and chlorpromazine). Values lower than 50% were considered low efflux pump inhibition activity (a), values between 50 and 100% were considered good efflux pump inhibition activity (b), whereas values above 100% were considered very good efflux pump inhibition activity (c). The result revealed that in *E. coli* (at ¼ MIC), compounds **LA1** and **LA2** had good to very good efflux pump inhibition activity with percentage inhibition of 87–111% and 86–110%, respectively (Table 6). In *P. aeruginosa* (both 1/4 and 1/2 MICs.), both compounds showed low efflux pump inhibition activity with values less than 50% (Table 7).

## 4. Discussion

The antimicrobial activity of the methanol-chloroform (4:1) extract of *L. africana* stem, three solvent fractions (petroleum ether, ethyl acetate, and methanol fractions), and some isolated phytoconstituents from the stem were investigated in this study. The effects of the crude extract and isolated compounds on bacterial biofilm formation and efflux pump activity were also studied. The resistance modulation effect of compounds on amoxicillin against *E. coli* and *P. aeruginosa* was also investigated.

Secondary metabolites, including those detected in this study, have been shown to have several biological actions, including antibacterial, anti-inflammatory, and antioxidant properties [17]. Terpenoids, phytosterols, and alkaloids have been reported to have antimicrobial, resistance modulation, antibiofilm, and efflux pump inhibition activities [17,22]. Tannins, flavonoids, and terpenoids have demonstrated antimicrobial, anti-inflammatory and antioxidant activities [22,23]. The antimicrobial effect of these secondary metabolites is elicited through various mechanisms. Phytochemicals such as terpenoids and phytosterols disrupt bacterial cell membrane through lipophilic action that leads to the disturbance of membrane-embedded proteins including efflux proteins, an increase in membrane permeability and fluidity and alteration of ion transport processes in both Gram-positive and Gram-negative bacteria [23]. Polyphenols such as flavonoids and tannins inhibit DNA and RNA synthesis in bacterial cells [23]. Therefore, the secondary metabolites found in *L. africana* may explain the traditional applications of the plant for treating a variety of ailments, including inflammatory disorders and infections.

The extracts and compounds demonstrated considerable antibacterial activities. Some authors [22,23,24] have proposed criteria for classifying the level of antimicrobial activity of plant extracts. Kuete et al. [22] classify the antimicrobial activity of plant extracts as significant (MIC < 100 µg/mL), moderate (100 ≤ MIC ≤ 625 µg/mL) or weak (MIC > 625 µg/mL). According to this criterion, the whole extract and fractions had moderate antibacterial activities. Generally, the extract and fractions had moderate antibacterial activities (MIC ≤ 500) against most of the organisms tested (Table 2). At MICs of 125 µg/mL, the whole extract showed activity against *S. aureus* ATCC 25923, *E. faecalis* ATCC 29212, *K. pneumoniae* (clinical strain), and *V. cholerae* (clinical strain). At MICs of 500 µg/mL, the whole extract showed activity against *E. coli* ATCC 25922, *P. aeruginosa* ATCC 27853, *S. typhi* (clinical strain), and *S. pyogenes* (clinical strain). The ethyl acetate fraction showed activity against *E. faecalis* ATCC 29212, *K. pneumoniae* (clinical strain), *V. cholerae* (clinical strain), and *P. mirabilis* ATCC 12453 at MICs of 250 µg/mL and against *S. typhi* (clinical strain) at 500 µg/mL. At MICs of 125 µg/mL and 250 µg/mL, the methanol fraction showed activity against *V. cholerae* (clinical strain) and *S. aureus* ATCC 25923, respectively. At MICs of 500 µg/mL, the petroleum ether fraction showed activity against *V. cholerae* (clinical strain) and *S. aureus* ATCC 25923. As a result, among the *L. africana* extract fractions, the ethyl acetate fraction demonstrated the most significant antimicrobial activity. However, the whole extract outperformed its fractions in terms of antimicrobial activity.

In the broth dilution assay, the compounds isolated from the stem of *L. africana* were tested for antimicrobial activity against Gram-positives (*S. aureus* and *E. faecalis*) and Gram-negatives (*P. aeruginosa* and *E. coli*). The compounds (**LA1** and **LA2**) showed antibacterial activity at MICs ranging from 31 to 125 g/mL (Table 4). The lower MICs of the isolated compounds compared with those of the parent fraction suggests that fractionation produced more active samples. The antimicrobial activity for pure compounds may be classified as significant (MIC < 10 µg/mL), moderate (10 ≤ MIC ≤ 100 µg/mL), or weak (MIC > 100 µg/mL) [22]. The friedelane triterpenoid (**LA1**) demonstrated moderate antibacterial activity whereas the phytosterol, β-sitosterol (**LA2**) showed weak to moderate antibacterial activity against the selected pathogens (Table 3). Triterpenoids and phytosterols have been reported to elicit their antibacterial effect through disruption of the cell membrane due to their lipophilic nature that leads to increased membrane fluidity, permeability, and disruption of embedded proteins [23]. It could therefore be speculated that the bulkier lipophilic nature of the friedelane triterpenoid (**LA1**) compared to the phytosterol, β-sitosterol (**LA2**) contributes to its higher activity over the β-sitosterol. The presence of these compounds in *L. africana* may thus contribute to its antibacterial activity.

According to the results of the resistance modulatory tests (Table 4), all compounds notably potentiated amoxicillin’s antibacterial activity against *E. coli* and *P. aeruginosa*. In the presence of the isolated compounds, the MIC of amoxicillin decreased from 320 µg/mL to 31.25 µg/mL, indicating a modulation factor (MF) of about 10 for *P. aeruginosa*. The MIC of amoxicillin in *E. coli* decreased from 20 µg/mL to 0.625 µg/mL, resulting in MF = 32. This means that when the compounds are co-administered with amoxicillin, the MIC of amoxicillin is about 10 times lower to elicit an antibacterial effect against *P. aeruginosa* and 32 times lower to elicit an antibacterial effect against *E. coli*. The findings indicate that the isolated compounds can enhance amoxicillin’s antibacterial effect against these bacteria.

Plant extracts and plant-derived compounds have been extensively studied for their resistance-modulatory effect on antibiotics [25,26,27,28,29,30]. According to some studies, the interaction of plant-derived compounds and antibiotics modulates bacterial resistance through bacterial membrane destruction, increased antibiotic influx into the bacterial cell, inhibition of bacterial efflux pumps, inhibition of quorum sensing, and gene expression modulation [31,32]. Triterpenoids have been reported to enhance the antibacterial activity of several classes of antibiotics including β-lactams, fluoroquinolones, tetracyclines, macrolides and glycopeptides [32]. Their mechanism of action is thought to be due to membrane disruption that enhances the influx of the antibiotics into the bacterial cell to reach high concentrations for maximum antibacterial action [32]. Amoxicillin is an example of a β-lactam antibiotic; hence, the resistance modulation action of the friedelane triterpenoid and β-sitosterol could be mediated by membrane destruction and the ability of the compounds to enhance the influx of the antibiotic into bacterial cells.

The whole extract of *L. africana* stem (LA) was tested for its ability to inhibit biofilm formation against *S. aureus* ATCC 25923, *E. coli* ATCC 29212, and *P. aeruginosa* ATCC 27853. The microorganisms used are known biofilm-forming pathogens [5]. The extract inhibited biofilm formation in a concentration-dependent manner between 15.6 and 500 µg/mL. The strongest biofilm inhibition effect was observed against *E. coli*, followed by *S. aureus* and *P. aeruginosa* (Figure 2). The percentage biofilm inhibition ranged between 40% and 59% for *S. aureus* at its sub-inhibitory concentration (15–62.5 µg/mL), and 49 and 77% for *E. coli* at its sub-inhibitory concentration (15–250 µg/mL). For *P. aeruginosa*, the percentage biofilm inhibition ranged between 15 and 56% at the sub-inhibitory concentration of 62.5–250 µg/mL whereas at concentrations between 15 and 31 µg/mL, LA enhanced the biofilm-forming capacity of *P. aeruginosa*. The effect of the triterpenoid compounds **LA1** and **LA2** (3.1- 100 µg/mL) on biofilm formation in *S. aureus*, *E. coli*, and *P. aeruginosa* is presented in Figure 3. **LA1** showed good antibiofilm activity against *S. aureus* (54–65%) and *E. coli* (47–74%) whereas it showed low to good activity against *P. aeruginosa* (42–57%). **LA2** demonstrated good antibiofilm activity against *S. aureus* (56–72%), *E. coli* (60–65%) and *P. aeruginosa* (50–65%).

A variety of plant extracts and compounds produced from plants have been shown to inhibit the formation of biofilms by blocking the adhesion or implantation of planktonic bacterial cells on abiotic surfaces [20,23]. Although this is the first report on the ability of *L. africana* and its friedelane-type triterpenoid (**LA1**) to inhibit biofilm formation, previous research has described the biofilm-inhibitory activity of a number of pentacyclic triterpenes and sterols, including β-sitosterol (**LA2**), which demonstrated remarkable anti-biofilm activities [16,32,33]. By destroying microbial membrane structures, blocking the synthesis of peptidoglycans, nucleic acids, quorum sensing, and anti-cell adhesion molecules, plant extracts and plant-derived chemicals may prevent the formation of biofilms [34,35,36,37].

The EtBr accumulation assay was used to test the ability of the crude extract (LA) and isolated compounds (**LA1** and **LA2**) to act as efflux pump inhibitors (EPIs). EtBr, a multidrug efflux pump substrate, emits a strong fluorescent signal when bound to DNA intracellularly but only a weak signal when present extracellularly. As a result of the retention of fluorescence over time if the efflux is reduced, the activity of putative EPIs can be measured fluorometrically [38]. The bacterial efflux pump phenomenon has been observed in *P. aeruginosa*, *E. coli*, and *S. aureus* [39]. *P. aeruginosa* and *E. coli* are classified as critical priority pathogens, whereas *S. aureus* is classified as a high-priority pathogen [40]. As generally known efflux pump expressors, *P. aeruginosa*, *E. coli*, and *S. aureus* were chosen for the efflux pump inhibitory effects.

Plant secondary metabolites, such as triterpenoids and sterols, have been found to block the action of bacterial efflux pumps [16]. **LA1** and **LA2** acted on efflux pumps as shown by the increased ethidium bromide accumulation in *E. coli* (Figure 5A,B) and *P. aeruginosa* (Figure 6A,B). Although their mechanism of action on efflux proteins is not clearly established, triterpenoids are reported to elicit efflux pump inhibition activity [41]. This suggests that the efflux pump inhibition effect demonstrated by **LA1** and **LA2** is due to their triterpenoid nature and thus contributes to the overall antibacterial activity of *L. africana*.

The antibacterial activity of *L. africana* and its constituents demonstrated in this study confirms the plant as a potential source of novel antibacterial agents alone and as adjuvants in combination with known antibiotics.

## 5. Conclusions

This work has established that *Loeseneriella africana* stem extract has antimicrobial resistance modulation, anti-biofilm generation, and efflux pump inhibitory activities. This lends scientific credence to the traditional usage of *L. africana* stem for treating infections. Friedelane-1,3-dione and β-sitosterol isolated from *L. africana* demonstrated an antibacterial resistance modulation effect, and antibiofilm and efflux pump inhibition activities in Gram-negative and Gram-positive organisms. The promising antimicrobial activities demonstrated by the constituents of *L. africana* further support the antimicrobial activities of the plant.

## Figures and Tables

**Table 1:** Phytochemical screening of the stem of *L. africana*.

Secondary Metabolite	Result
Reducing sugars	+
Tannins	+
Flavonoids	+
Coumarins	+
Triterpenoids	+
Phytosterols	+
Saponins	−
Alkaloids	+

+: detected; −: not detected.

**Table 2:** 1H and 13C NMR data for compounds **LA1** and **LA2**.

Position	LA1		LA2	
	δ C	δ H (*J in Hz*)	δ C	δ H (*J in Hz*)
1	202.8 (C)	-	37.2 (CH_2_)	1.81
2	60.6 (CH_2_)	3.21, *d* (15.9 Hz) 3.43, *d* (15.9 Hz)	31.6 (CH_2_)	1.81
3	204.2 (C)	-	71.8 (CH)	3.50
4	59.0 (CH)	2.55, *q* (6.7 Hz)	42.2 (CH_2_)	2.21, 2.27
5	37.8 (C)	-	140.7 (C)	-
6	40.5 (CH_2_)	1.35, *m* 1.86 *m* (12. 5, 6.8, 3.2 Hz)	121.7 (CH)	5.33, *d* (4.7 Hz)
7	18.0 (CH_2_)	1.42, 1.51	31.9 (CH_2_)	1.55, 1.56
8	52.1 (CH)	1.22	31.9 (CH)	1.42
9	37.13 (C)	-	50.1 (CH)	0.89
10	71.79 (CH)	2.35, *s*	36.5 (C)	-
11	34.5 (CH_2_)	1.12, *m* 2.12, *m* (13.6, 6.8, 3.5 Hz)	21.0 (CH_2_)	1.44, 1.47
12	30.1 (CH_2_)	1.26, 1.38	39.7 (CH_2_)	1.13, 1.99
13	39.4 (C)	-	42.2 (C)	-
14	38.2 (C)	-	56.7 (CH)	0.96
15	32.3 (CH_2_)	1.50, *m*	24.3 (CH_2_)	1.03, 1.56
16	35.8 (CH_2_)	1.33, 1.52	28.2 (CH_2_)	1.26, 1.81
17	30.0 (C)	-	56.0 (CH)	1.08
18	42.6 (CH)	1.54, *m*	11.8 (CH_3_)	0.65 s
19	35.2 (CH_2_)	1.32, *m*	19.4 (CH_3_)	0.98, s
20	28.1 (C)	-	36.1 (CH)	1.32, *m*
21	32.7 (CH_2_)	1.24, *m*	18.7 (CH_3_)	0.90, *d* (6.5 Hz)
22	39.2 (CH_2_)	0.90, 1.47	33.9 (CH_2_)	0.97, 1.30, *dd* (8.4, 15.0 Hz)
23	7.3 (CH_3_)	1.02, *d* (6.7 Hz)	26.0 (CH_2_)	1.13, *dd* (8.4, 15.0 Hz)
24	15.9 (CH_3_)	0.66, *s*	45.8 (CH)	0.90
25	18.0 (CH_3_)	1.17, *s*	29.1 (CH)	1.65
26	20.3 (CH_3_)	1.00, *s*	19.0 (CH_3_)	0.81, *d* (2.5 Hz)
27	18.7 (CH_3_)	0.99, *s*	19.8 (CH_3_)	0.79, *d* (6.8 Hz)
28	32.0 (CH_3_)	1.15, *s*	23.0 (CH_2_)	1.21, 1.25
29	31.7 (CH_3_)	0.97, *s*	12.0 (CH_3_)	0.82, *t* (6.0 Hz)
30	35.0 (CH_3_)	0.91, *s*	22.3 (CH_2_)	0.84, *m*

**Table 3:** MICs of *L. africana* stem extract and fractions against clinically significant bacteria in HT-SPOTi assay.

Microorganism	Minimum Inhibitory Concentration (µg/mL)				
	LA	LAPE	LAEt	LAM	Amox
*S. aureus*	125	500	>500	250	3.91
*S. pyogenes*	500	>500	>500	>500	1.95
*E. faecalis*	125	>500	250	>500	0.49
*P. aeruginosa*	500	>500	>500	>500	500
*P. mirabilis*	>500	>500	250	>500	31.25
*K. pneumoniae*	125	>500	250	>500	31.25
*S. typhi*	500	>500	500	>500	62.50
*E. coli*	500	>500	>500	>500	125
*V. cholerae*	125	500	250	125	125

Key: LA—whole extract, LAPE—Pet. ether fraction, LAEt—Ethyl acetate fraction, LAM—Methanol fraction, and Amox—Amoxicillin.

**Table 4:** MICs of isolated compounds from *L. africana* stem extract.

Microorganism	Minimum Inhibitory Concentration (µg/mL)		
	LA1	LA2	Amoxicillin
*S. aureus*	31.25	31.25	10
*E. faecalis*	31.25	31.25	10
*E. coli*	62.5	125	20
*P. aeruginosa*	62.5	125	>320

**Table 5:** Minimum inhibitory concentration (MIC) of amoxicillin in the absence or presence of compounds at ¼ MIC concentration.

Microorganism	MIC (µg/mL)	MIC Combined (µg/mL)		Modulation Factor	
	Amoxicillin Only	LA1	LA2	LA1	LA2
*P. aeruginosa*	>320	<31.25	<31.25	>10	>10
*E. coli*	20	<0.625	<0.625	>32	>32

**Table 6:** Effect of *L. africana* compounds on *E. coli* ATCC 25922 efflux pump activity expressed as a percentage.

MIC	Compound	% Inhibition of Efflux Pump Activity			
		15 min	30 min	45 min	60 min
1/4	**LA1**	111.0 ± 19.03 ^c^	108.4 ± 15.43 ^c^	100.0 ± 11.05 ^c^	87.1 ± 12.47 ^b^
	**LA2LA2**	109.6 ± 12.89 ^c^	105.4 ± 12.17 ^c^	95.4 ± 12.82 ^c^	86.3 ± 9.34 ^b^
1/2	**LA1**	59.5 ± 11.35 ^b^	45.3 ± 8.01 ^a^	34.6 ± 5.00 ^a^	29.1 ± 1.78 ^a^
	**LA2**	61.3 ± 12.55 ^b^	50.8 ± 6.35 ^b^	39.2 ± 12.55 ^a^	30.7 ± 7.10 ^a^
	Verapamil	60.3 ± 5.41 ^b^	120.3 ± 6.82 ^c^	125.8 ± 7.23 ^c^	113.4 ±8.29 ^c^
	Chlorpromazine	95.6 ± 7.91 ^b^	97.9 ± 15.04 ^b^	78.64 ± 5.78 ^b^	71.9 ± 4.49 ^b^

Key: superscripts (a, b, and c) represent low, good, and very good efflux pump inhibition activity, respectively, expressed over the negative control. Data presented as mean ± SEM (*n* = 3); *p* < 0.0001.

**Table 7:** Effect of *L. africana* compounds on *P. aeruginosa* ATCC 27853 efflux pump activity expressed as a percentage.

MIC	Compound	% Inhibition of Efflux Pump Activity			
		15 min	30 min	45 min	60 min
1/4	**LA1**	35.3 ± 26.88 ^a^	18.1 ± 10.87 ^a^	6.7 ± 10.69 ^a^	14.5 ± 14.21 ^a^
	**LA2**	6.7 ± 5.34 ^a^	2.2 ± 7.66 ^a^	0.5 ± 8.55 ^a^	8.7 ± 5.27 ^a^
1/2	**LA1**	41.7 ± 10.06 ^a^	19.3 ± 11.08 ^a^	13.22 ± 9.91 ^a^	13.4 ± 0.28 ^a^
	**LA2**	30.6 ± 11.04 ^a^	26.2 ± 8.94 ^a^	29.2 ± 11.20 ^a^	37.1 ± 9.67 ^a^
	Verapamil	147.3 ± 28.78 ^c^	145.5 ± 17.35 ^c^	129.3 ± 7.34 ^c^	128.4 ±10.89 ^c^
	Chlorpromazine	105.4 ± 9.02 ^c^	107.6 ± 9.03 ^c^	86.0 ± 3.86 ^b^	85.9 ± 9.91 ^b^

Key: superscripts (a, b, and c) represent low, good, and very good efflux pump inhibition activity, respectively, expressed over the negative control. Data presented as mean ± SEM (*n* = 3); *p* < 0.0001.

## Data Availability

The dataset supporting the conclusions of this article are included within the article and its additional files. Raw data sets used and analysed during the study are available from the corresponding author on reasonable request.

## Supplementary Materials

The following supporting information can be downloaded at: https://www.mdpi.com/article/10.3390/microorganisms12010007/s1, Figure S1: Schematic presentation of isolation procedure; Figure S2: (A) Structure of **LA1** (B) 1H-1H COSY and crucial 1H-1H NOE correlations of **LA1** (C) Crucial HMBC correlations of **LA1**; Figure S3: Structure of **LA1** (A) rotated horizontally at 180° (B), then further rotated in a plane counterclockwise at 60 °C (D) Structure of **LA1** numbered according to the position presented in Table 1; Figure S4: (A) Structure of **LA2** (B) 1H-1H COSY and crucial 1H-1H NOE correlations of **LA2** (C) Crucial HMBC correlations of **LA2**.
