# Supplementary material for: Antibacterial, Resistance Modulation, Anti-Biofilm Formation, and Efflux Pump Inhibition Properties of Loeseneriella africana (Willd.) N. Halle (Celastraceae) Stem Extract and Its Constituents
Source: Microorganisms. 2023 Dec 19;12(1):7. doi: 10.3390/microorganisms12010007 (PMC10819663; doi:10.3390/microorganisms12010007)
Supplement: Supplementary file 1 [file microorganisms-12-00007-s001.zip › microorganisms-2725832-supplementary.pdf]

Antibacterial, Resistance Modulation, Anti-Biofilm Formation, and Efflux Pump Inhibition Properties of *Loeseneriella africana* (Willd.) N. Halle (Celastraceae) Stem Extract and Its Constituents

Daniel Anokwah [1*], Evelyn Asante-Kwatia [2], Jonathan Asante [1], Daniel Obeng-Mensah [1], Cynthia Amaning Danquah [3], Isaac Kingsley Amponsah [2], Elvis Ofori Ameyaw [1], Robert Peter Biney [1], Ernest Obese [1], Lukas Oberer [4], Daniel Gyamfi Amoako [5], Akebe Luther King Abia [5,6] and Abraham Yeboah Mensah [2]

[1] School of Pharmacy and Pharmaceutical Sciences, College of Health and Allied Sciences, University of Cape Coast, PMB, Cape Coast, Ghana; jonathan.asante@ucc.edu.gh (J.A.); daniel.obeng-mensah@ucc.edu.gh (D.O.M.); eameyaw@ucc.edu.gh (E.O.A.); robert.biney@ucc.edu.gh (R.P.B.); ernest.obese@ucc.edu.gh (E.O.)

[2] Department of Pharmacognosy, Faculty of Pharmacy and Pharmaceutical Sciences, Kwame Nkrumah University of Science and Technology, PMB, Kumasi, Ghana; eamireku@knust.edu.gh (E.A.-K.); akila.amponsah@gmail.com (I.K.A.); aymensah@yahoo.com (A.Y.M.)

[3] Department of Pharmacology, Faculty of Pharmacy and Pharmaceutical Sciences, Kwame Nkrumah University of Science and Technology, PMB, Kumasi, Ghana; cadanq@yahoo.com

[4] Novartis Institutes for BioMedical Research CH-4056, Basel, Switzerland; lukas.oberer@bluewin.ch

[5] Antimicrobial Research Unit, College of Health Sciences, University of KwaZulu-Natal, Durban 4001, South Africa; amoakodg@gmail.com (D.G.A.); lutherkinga@yahoo.fr (A.L.K.A.)

[6] Environmental Research Foundation, Westville 3630, South Africa

* Correspondance : daniel.anokwah@ucc.edu.gh (D.A.)

SUPPLEMENTARY MATERIAL

Protocol S1: Details of the isolation and characterization of bioactive constituents from *Loeseneriella africana* stem

About 3kg of *Loeseneriella africana* stem powder was extracted with methanol-chloroform (4:1) and the extract was concentrated under reduced pressure and temperature with a rotary evaporator and further dried at 60 °C in an oven to afford a brown solid extract (LAE). About 100 g of the LAE was partitioned into three fractions with petroleum ether, ethyl acetate (EtOAc) and methanol (Figure 1). About twenty-five grams (24.6 g) of the EtOAc fraction was adsorbed onto Si gel 60, loaded onto previously packed Si gel 60 column and separation was done by gradient elution method with Pet. ether, EtOAc and MeOH in increasing order of polarity. Eighty-one (81) fractions were collected in volumes of 250 mL and bulked into fractions (F1 – F6) using TLC as a guide. Purification of fraction F2 (2.8 g) afforded 31 fractions of 30 mL volume each. These were bulked into 2 sub fractions (F2i- ii) using their TLC profile. F2i was repeatedly washed with methanol to obtain compounds LA1 (26 mg). Purification of fraction F3 (3.1 g) afforded 35 fractions of 30 mL volume each. These fractions were bulked into 3 sub fractions (F3i-iii) using their TLC profile. F3i and F3ii were washed repeatedly washed with petroleum ether and methanol to obtain compound LA2 (26 mg) and LA3 (31 mg) respectively

Compound LA1 and LA2 were found to be the same from the TLC fingerprint, hence combined and labelled as LA1 whiles LA3 was labelled as LA2 for further analysis. All isolates were kept in refrigerator until required for characterization. The isolation process is elaborated with a schematic presentation (Figure S1).

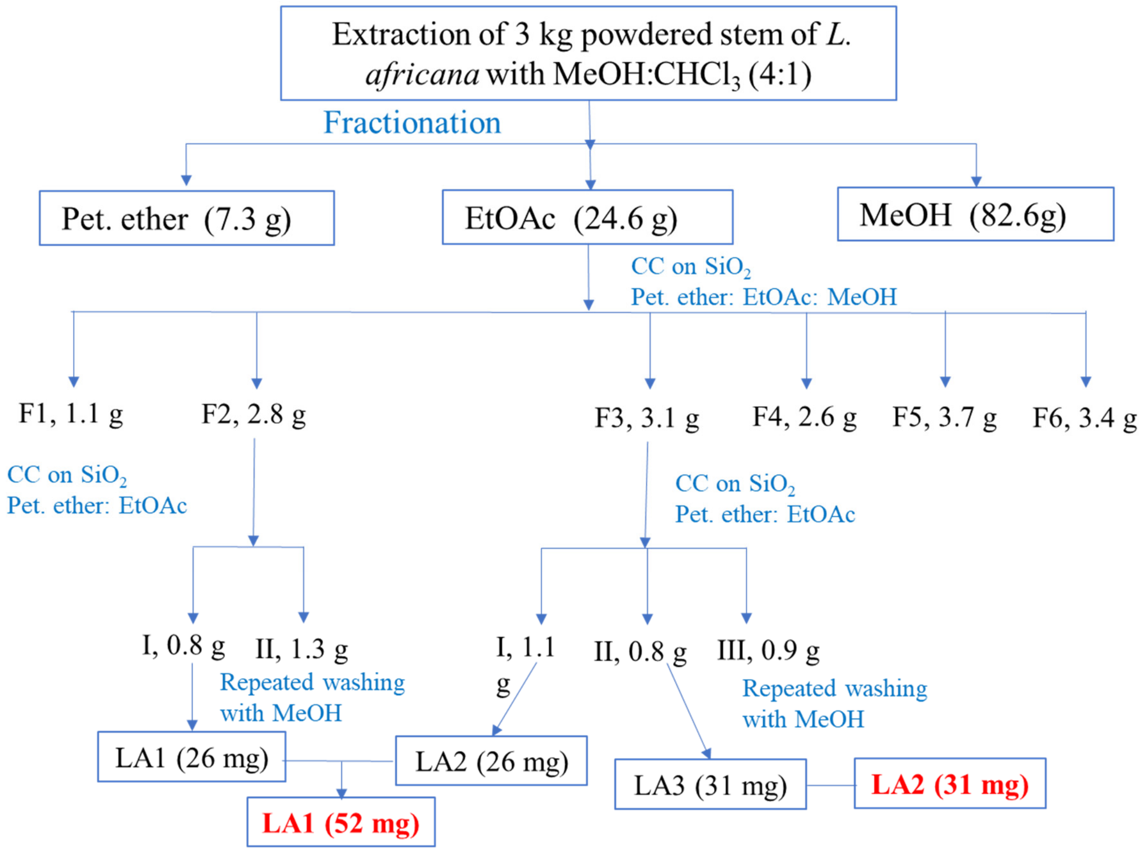

Figure S1. Schematic presentation of isolation procedure.

Result

A comparison of the ^{1}H and ^{13}C NMR data with the literature was used to identify the compounds LA1 and LA2.

Compound LA1 (friedelane-1,3-dione)

White amorphous crystals: ^{1}H NMR (600 MHz in $CDCl_3$) and ^{13}C (125 MHz in $CDCl_3$) NMR data are shown in Table 1

Its molecular formula was deduced as $C_{30}H_{48}O_2$ from its positive molecular ion peak at m/z of 441.372819 $[M + H]^+$ ($C_{30}H_{49}O_2$) and its [M – H], m/z of 439.357732 ($C_{30}H_{47}O_2$) in the HRESI-MS data (appendix 29 C) suggesting seven ring and double bond equivalents. The FTIR spectrum of LA1 showed absorption bands at 1730 cm^{-1} and 1703 $cm^{-1,}$ which are characteristic of a diketone.

The ^{13}C NMR data (Table 1) showed signals for 30 carbons data showed the presence of four methine, ten methylene, eight methyl and eight quaternary carbons (including two carbonyls). The ^{1}H and ^{13}C assignment were verified with data from the HSQC spectra and the connectivity was established with data from the HMBC and ^{1}H-^{1}H COSY spectra. Careful analysis of the ^{13}C NMR data revealed characteristic signals for a diketone [δ_C 202.8 (C-1) and 204.2 (C-3)], one de-shielded methylene [δ_C 60.6 (C-2)], two de-shielded methine groups [δ_C 59.0 (C-4) and δ_C 71.8 (C-10)] and eight tertiary methyl groups (Table 4.39). The ^{1}H NMR spectrum gave signals for eight methyl protons [δ_H 1.02, *d*, (H_3-23), δ_H 0.66, *s*, (H_3-24), 1.17, *s*, (H_3-25), δ_H 1.00, *s*, (H_3-26), δ_H 0.99, *s*, (H_3-27), δ_H 1.15, *s*, (H_3-28), δ_H 0.97, *s*, (H_3-29) and δ_H 0.91, *s*, (H_3-30)], two de-shielded methylene protons [δ_H 3.21, *d*, (H-2a) and 3.43, *d*, (H-2b)] and two de-shielded methine protons [δ_H 2.55, *q*, (H-4) and δ_H 2.35, *s*, (H-10)]. The ^{1}H NMR revealed an AB system at δ 3.21 and 3.43 (J = 15.9 Hz) assigned to the methylene protons between the two carbonyls of the diketone. HMBC correlations of the protons of H-2 with methine carbons [δ_C 59.0 (C-4) and δ_C 71.8 (C-10)] and with carbonyl carbons [δ_C 202.8 (C-1) and δ_C 204.2 (C-3)] confirmed the presence of a 1-3 diketone which is possible in the ring A of a triterpenoid. The position of the ketone group at position 3 was verified with the HMBC correlation between methyl protons at δ_H 1.02 (H_3-23) and the carbonyl carbon at δ_C 204.2 (C-3). The position of the methyl group at C-5 was verified from the HMBC correlations of the methyl protons at δ_H 0.66 (H_3-24) with carbons at δ_C 59.0 (C-4), δ_C 37.8 (C-5), δ_C 40.5 (C-6) and δ_C 71.8 (C-10). HMBC correlations of methyl protons at δ_H 1.15 (H_3-28) with δ_C 35.8 (C-16), δ_C 30.0 (C-17), δ_C 42.6 (C-18) and δ_C 39.2 (C-22) confirmed the presence of methyl group at C-17. Similarly, the positions of the other methyl groups were verified with the HMBC correlations (Figure 2 C). Based on data from ^{1}H-^{1}H NOE correlations (Figure S2 B), H-8, H-10, CH3-27 and CH3-30 were designated β-oriented, whereas CH3-23, CH3-24, CH3-25, CH3-26, CH3-28 and CH3-29 were designated as α-oriented.

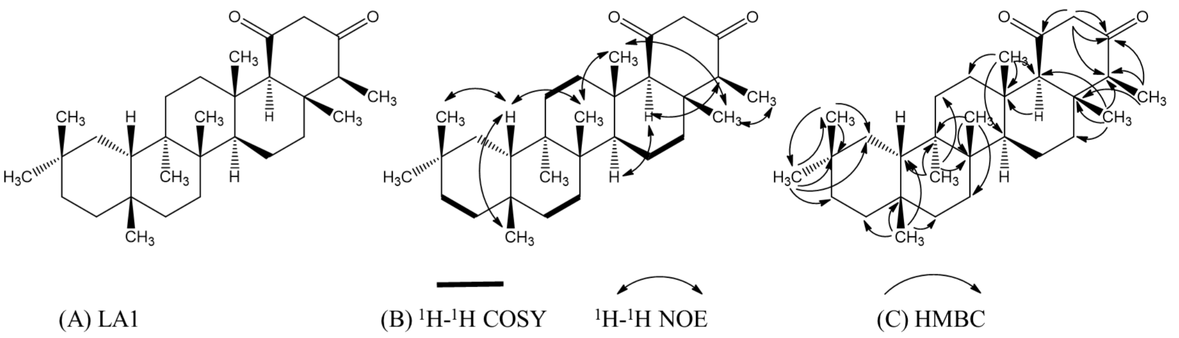

Figure S2 (A) Structure of LA1 (B) ^{1}H-^{1}H COSY and crucial ^{1}H-^{1}H NOE correlations of LA1 (C) Crucial HMBC correlations of LA1.

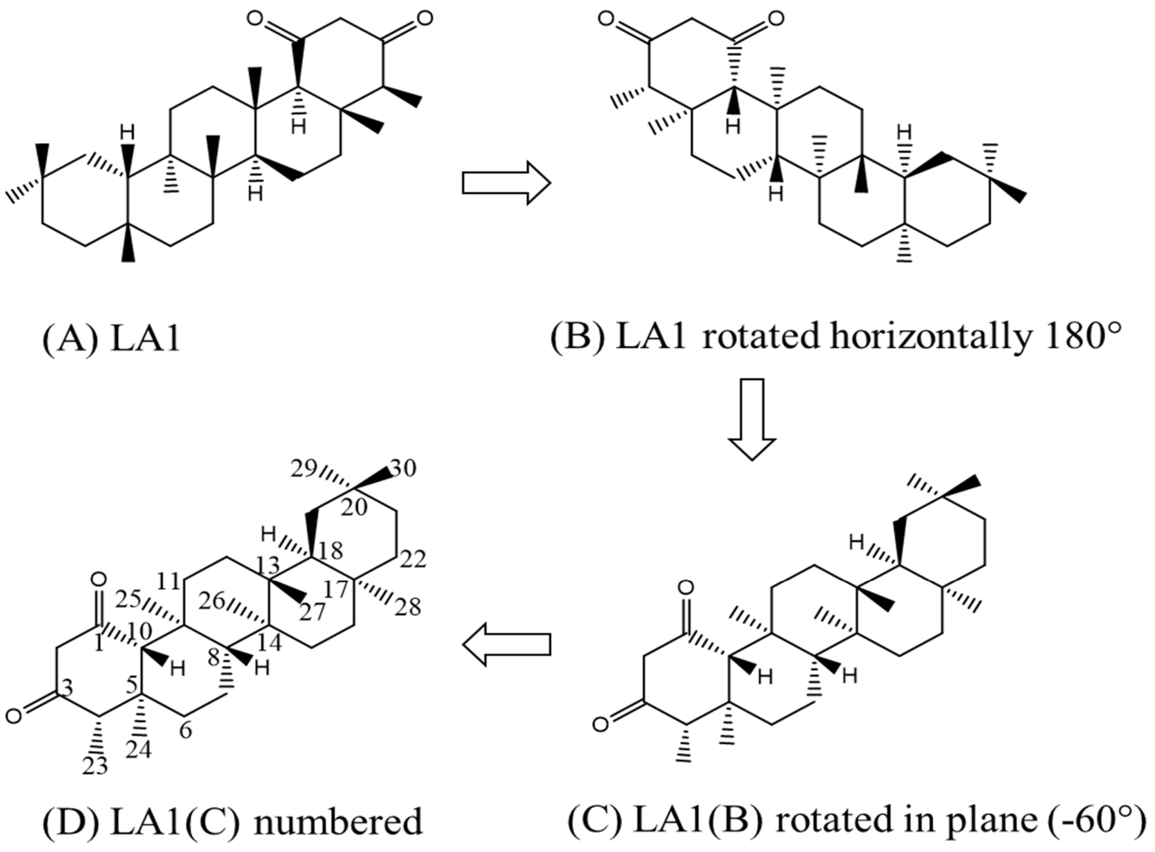

Figure S3: Structure of LA1 (A) rotated horizontally at 180º (B), then further rotated in a plane counterclockwise at 60º(C) (D) Structure of LA1 numbered according to the position presented in Table 1

Compound LA2 (β-sitosterol)

White amorphous powder; positive EI–MS *m/z* 414.7067 $[M]^+$ (calcd. for $C_{29}H_{50}O$, 414.3849). ^{1}H NMR (600 MHz in $CDCl_3$) and ^{13}C (125 MHz in $CDCl_3$) NMR data are shown in Table 1. The FTIR `data for LA2; OH (broad band at 3403 cm^{-1}), alcoholic C-O stretching at 1176 cm^{-1} and C-H stretch for alkene at 2956 cm^{-1}

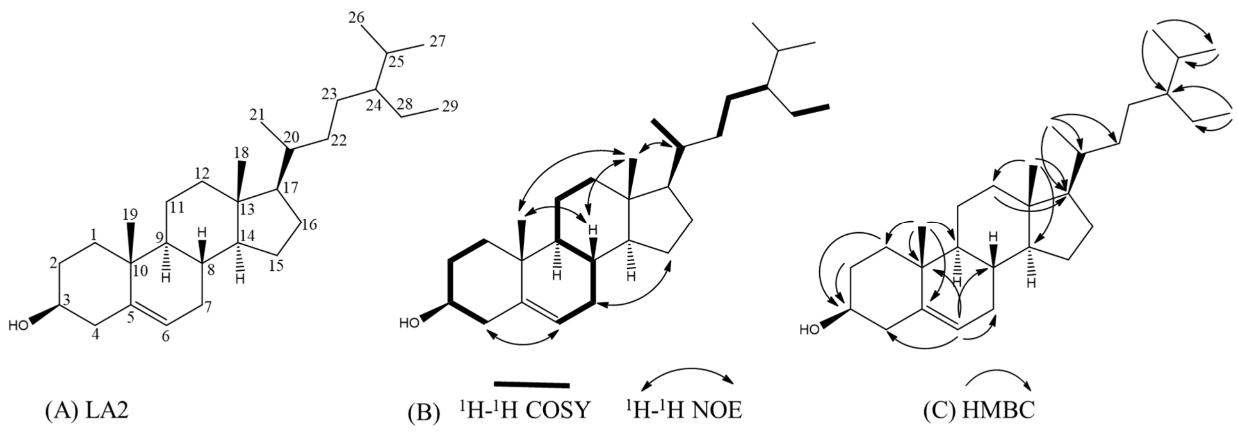

Figure S4: (A) Structure of LA2 (B) 1H-1H COSY and crucial 1H-1H NOE correlations of LA2 (C) Crucial HMBC correlations of LA2
